# Bat assemblages from three Atlantic Forest fragments in Rio de Janeiro state, Southeastern Brazil

**DOI:** 10.3897/BDJ.3.e4404

**Published:** 2015-01-19

**Authors:** Roberto Leonan Morim Novaes, Daniel Tavares Cassilhas Rosa, Davor Vrcibradic, Leonardo dos Santos Avilla

**Affiliations:** † Fundação Oswaldo Cruz, Rio de Janeiro, Brazil; ‡ Universidade Federal do Rio de Janeiro, Rio de Janeiro, Brazil; § Universidade Federal do Estado do Rio de Janeiro, Rio de Janeiro, Brazil

**Keywords:** Atlantic Forest remnants, Chiroptera, Neotropical bats, species richness.

## Abstract

Bat species richness in Neotropical localities is generally higher than that of any other group of mammals, and surveys of local bat assemblages may provide useful data for conservation management plans. Although the bat fauna of the Rio de Janeiro state is currently one of the best known in Brazil, there are several localities not adequately surveyed yet, and most of them are in the mountainous regions and in the northern portion of the state. From January 2008 to November 2009, we conducted surveys of bats in three localities in the state of Rio de Janeiro (municipalities of Varre-Sai, Sumidouro, and Cantagalo), and our fieldwork constitutes the first assessment of the bat assemblages of these localities. Surveys were conducted using mist nets in four different habitat types in each locality (forest interior, forest edge, riparian forest, and open areas [pastures]). We captured a total of 148 individuals in 17 species, 14 genera and 3 families. Among them, 11 species were recorded in Sumidouro, seven in Cantagalo, and nine in Varre-Sai. Although species richness was low compared with previous surveys in other close localities, we recorded species that have been rarely sampled in Southeastern Brazil (e.g., *Macrophyllum macrophyllum* [Phyllostomidae]). The results reinforce the importance of sampling different habitats in short surveys to improve the number of species registered.

## Introduction

The Brazilian Atlantic Forest is one of the most endangered biomes on Earth (Ribeiro et al. 2009), and due to its high biodiversity and rates of endemism it is considered one of the world’s hotspots, and an area of high priority for conservation (Myers et al. 2000). Because of a historical process of exploiting the land for timber, cultivation of coffee, sugar cane, and, more recently, pastures for livestock (Galindo-Leal and Câmara 2005), the original vegetation was reduced to about 11%, and most of the remnant vegetation is currently diffused in small fragments of second-growth forests (Ribeiro et al. 2009). Biodiversity surveys in these remnants are necessary to subsidize conservation management plans for local faunas and floras. Also, the study of local faunas is essential for understanding the regional patterns of biological diversity, and allows better characterizations of the geographic distribution of specific taxa (Soulé and Wilcox 1980).

Bats represent the second most speciose mammalian order, and can make up more than half of the mammal species in some Neotropical communities (Tim 1994). Surveys of bat faunas can be important tools to evaluate the degree of habitat conservation in forest remnants (Medellín et al. 2000, Estrada and Coates-Estrada 2002). According to Esbérard (2003) the richness and diversity of bat species depends on the local availability of food and shelter, so there is a relationship between bat community composition and complexity of habitats available within a given site (Estrada et al. 1993, Sedlock et al. 2008). Thus, different habitats should be sampled to adequately survey local faunas (Kunz and Kurta 1988), including both human-modified and natural environments.

The state of Rio de Janeiro has one of the best studied bat faunas in Brazil (Bergallo et al. 2003, Esbérard and Bergallo 2005, Bernard et al. 2010). However, the mountainous region (i.e., slopes of Serra do Mar and Serra da Mantiqueira) and the northern portion of the state still represent gaps in the knowledge of the bat fauna, and surveys are highly required (Moratelli and Peracchi 2007, Modesto et al. 2008a, Modesto et al. 2008b, Esbérard et al. 2010, Peracchi and Nogueira 2010). Here, we present lists of bat species from three previously unsampled localities in the state of Rio de Janeiro (two in mountainous areas and one in the extreme north of the state), with comments on the importance of sampling in different habitats.

## Material and methods

### Study areas

The study was conducted in the municipalities of Varre-Sai, Sumidouro, and Cantagalo (Fig. 1). The three areas are highly fragmented due to the historical process of land use for agriculture and cattle ranching, and they represent gaps in the knowledge of occurrence of mammals, in general, and bats, in particular, for the state of Rio de Janeiro (Bergallo et al. 2009).

Varre-Sai is in the extreme north of the state of Rio de Janeiro, bordering the state of Espírito Santo. The locality sampled is situated at the Serra da Sapucaia (20°55'50''S, 41°53'54''W; altitude ca. 800 m), an extension of the Serra do Caparaó, in the Mantiqueira range. Sampling was carried out in fragments of seasonal semideciduous forest surrounded by matrix of pastures, agricultural fields and coffee plantations.

Sumidouro is in the mountains of the central region of Rio de Janeiro, at the continental border of the Serra do Mar. Fieldwork was carried out in a small fragment of dense ombrophilous forest on top of an inselberg-type rock formation named Pedra de Santa Rita (22°07'38"S, 42°41'00"W; altitude ca. 900–1000 m), an area with several natural caves.

Cantagalo is also in the mountains of the central region of the state of Rio de Janeiro, in the north of the continental border of Serra do Mar. Samplings were carried out in the Novo Tempo cave and surrounding areas (21°48'53"S, 42°11'57"W; altitude ca. 400 m), in a region of dense ombrophilous forest. The Novo Tempo cave is one of the largest caves in the state of Rio de Janeiro, and is located in a region formed by a mosaic of secondary forest fragments of various sizes, agricultural fields and open areas (pastures).

### Data collecting and analysis

Bat surveys were conducted from January 2008 to November 2009, with one sampling in the dry season and other in the rainy season in each locality. Each sampling was carried out from two to five nights. Bats were collected using mist nets (9x3 m, 25 mm-dash) placed on trails inside forested areas, at the edges of forest fragments, at the margins or over water bodies, and at the entrances or inside natural cavities that bats were using as roosts (Kunz and Kurta 1988). We used four to eight mist nets from sunset to sunrise. Sampling effort was calculated following Straube and Bianconi (2002), and resulted in a total of 6,480m²/h for Varre-Sai (five nights), 8,100m²/h for Sumidouro (seven nights) and 4,860m²/h for Cantagalo (four nights), with an almost equal effort for the four different habitats types (ca. 3,760m²/h). The following habitats were sampled: (1) forest, with mist nets placed inside three forest fragments of 54, 91 and 122 ha; (2) edges of these same fragments; (3) riparian forest, with mist nets placed in the margin or perpendicular to watercourses; and (4) open areas, with mist-nets placed in the pastures, which were 50 to 80 m far from the fragments. These four habitat types were present in the three sampled localities.

Bats captured were measured, sexed and identified in the field. Identifications followed Vizotto and Taddei (1973), Simmons and Voss (1998),Dias et al. (2002) and Dias and Peracchi (2008). Voucher specimens of all species per locality were collected and deposited in the collection of mammals of the Museu Nacional, Rio de Janeiro (MN), and collection of bats of the Universidade Federal Rural do Rio de Janeiro (LDM [see Data resources]).

Assemblages were compared by locality and habitat using the diversity index of Shannon-Wiener, and equitability and dominant species index (Magurran 1998). A rarefaction curve (95% confidence) was produced using the PAST software (Hammer et al. 2001), and the capture efficiency was calculated dividing the total captures by the sampling effort.

## Data resources

Voucher specimens were deposited at the mammal collection of Museu Nacional, Rio de Janeiro (MN) and at the bat collection of Laboratório de Diversidade de Morcegos (LDM), Universidade Federal Rural do Rio de Janeiro, Rio de Janeiro.

Sumirouro, Rio de Janeiro, Brazil: *Anoura caudifer* (MN 77690-77695, 77710, 77711-77713, 77715, 77716, 77718-77720); *Artibeus lituratus* (MN 77696, 77700, 77701); *Artibeus obscurus* (MN 77698, 77717); *Carollia perspicillata* (MN 77699, 77702, 77706, 77714, 77732); *Chiroderma doriae* (LDM 5266, 5267); *Chrotopterus auritus* (MN 77726, 77729, 77730); *Desmodus rotundus* (MN 77697, 77708, 77727, 77731, 77733); *Diphylla ecaudata* (MN 77728); *Macrophyllum macrophyllum* (MN 77735); *Platyrrhinus lineatus* (MN 77703); *Platyrrhinus recifinus* (MN 77705, 77709); *Vampyressa pusilla* (MN 77704, 77707). Varre-Sai, Rio de Janeiro, Brazil: *Artibeus fimbriatus* (MN 77725); *Carollia perspicillata* (MN 77722, 77723); *Myotis nigricans* (MN 77724); *Platyrrhinus lineatus* (MN 77734); *Sturnira lilium* (MN 77721). Cantagalo, Rio de Janeiro, Brazil: *Carollia perspicillata* (MN 77738, 77746, 77749); *Desmodus rotundus* (MN 77739-41, 77743, 77745); *Diphylla ecaudata* (MN 77747, 77748); *Glossophaga soricina* (MN 77736); *Peropteryx macrotis* (MN 77737, 77742, 77744, 77750-77752).

## Results and Discussion

We captured a total of 148 bats of 17 species for the three localities together (Sumidouro = 82 individuals of 11 species; Cantagalo = 25 individuals of seven species; Varre-Sai = 41 individuals of nine species). *Carollia perspicillata* was the most frequent species in the three areas, representing more than 50% of all bats recorded at Varre-Sai and more than 35% of the records from the other two areas (Table 1). *Carollia perspicillata* seems to be the dominant species in most Atlantic Forest localities in Rio de Janeiro (see Esbérard et al. 2006, Dias et al. 2008, Luz et al. 2011, Delciellos et al. 2012), and one of the most common in the Neotropics.

Individuals of frugivorous bats accounted for most of the captures in the three areas, and in the four habitats sampled as well. The dominance of frugivorous species typical of forest edge, such as *C. perspicillata*, is common in secondary forest fragments and agricultural areas (Heithaus and Fleming 1978, Faria 2006, Rocha et al. 2010, Novaes et al. 2014), since these species are benefited by the presence of pioneer vegetation and by high fruit production (Herrera et al. 1994, Murcia 1995, Guariguata and Sáenz 2002, Asbjornsen et al. 2004). The use of mist-nets may biased the low sampling of insectivorous bats, becuase it is more efficient for sampling representatives of phytophagous species (such as stenodermatines and carolliines) than animalivorous species, which can detect mist-nets easily (Portfors et al. 2000, Gorresen et al. 2008).

The three areas sampled had similar values for species diversity, equitability and dominance (Table 1). However, there were significant differences between these indices when compared by habitat. The forest interior showed higher species richness and species diversity (H’=2.27) than the other habitats, but there were no dominance (D’=0.12) (Table 2), indicating that the forest interior is subject to less environmental stress than the other habitats. The forest interior also had a high evenness of species composition (J’=0.91), and a greater number of exclusive species (five) when compared with the other habitats.

The higher species richness within the forested areas was expected, since these environments have more heterogeneous habitats, allowing coexistence of more species from different trophic guilds than other habitats, including those species with more specialized feeding habits (Kalko et al. 1996). Nevertheless, five species were recorded neither in the interior nor at the edge of forests, among them: *Peropteryx macrotis* and *Glossophaga soricina* were collected only in open areas; *Macrophyllum macrophyllum* and *Platyrrhinus lineatus* were collected only near or over water bodies in riparian forest; and *Myotis nigricans* was collected both in open areas and riparian forest. These observations reinforce the importance of sampling different habitats during short-term species surveys.

The three regions sampled showed low richness and diversity of species compared to other studies carried out in mountainous areas and other close localities (e.g., Esbérard 2007, Moratelli and Peracchi 2007, Dias et al. 2008, Esbérard et al. 2010). This may be due to the low sampling effort employed in the present study, when compared with the aforementioned studies, or even due to the characteristics of the landscape, since most of the previous studies conducted in mountainous regions of Rio de Janeiro were concentrated in areas of continuous forest, which support higher species richness (Medellín et al. 2000). Therefore, it is possible that the high degree of human-induced disturbance and habitat fragmentation in these areas have resulted in the loss of more sensitive species.

Even considering the low species richness, the areas sampled in the present study yielded some interesting records. An individual of *Macrophyllum macrophyllum*, a species considered rare in the state of Rio de Janeiro, was captured in a mist-net placed over water bodies, in Sumidouro. According to Weinbeer et al. (2005), this species has a strong association with habitats with collections of water, since it forages close to the water, catching aerial insects or “fishing” semi-aquatic insects from the water surface (Meyer et al. 2005).

We known very little about bats inhabiting caves in Southeastern Brazil. Captures with mist-netsset up inside the Novo Tempo cave revealed colonies of *Peropteryx macrotis*, *Desmodus rotundus*, *Diphylla ecaudata*, *Carollia perspicillata* and *Artibeus obscurus*.

**Figure 2.**
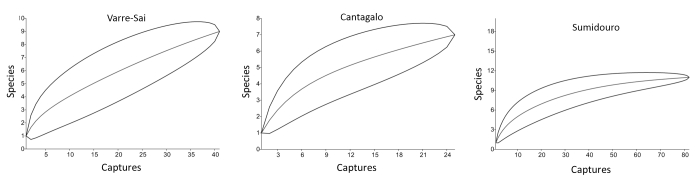
Species rarefaction curve for the three bat assemblages in Rio de Janeiro, southeastern Brazil (see Suppl. material 1).

Considering the three areas combined, the list of species obtained here is still very preliminary, with the species accumulation curve did not reaching an asymptote (Fig. 2). However, the results of this study indicate that sampling different habitats within a given locality increase the efficiency of bat inventories, in particular, during short surveys.

## Supplementary Material

Supplementary material 1 Data of rarefaction curve Data type: table
File: oo_36087.csv Roberto Leonan Morim Novaes

## Figures and Tables

**Figure 1a.**
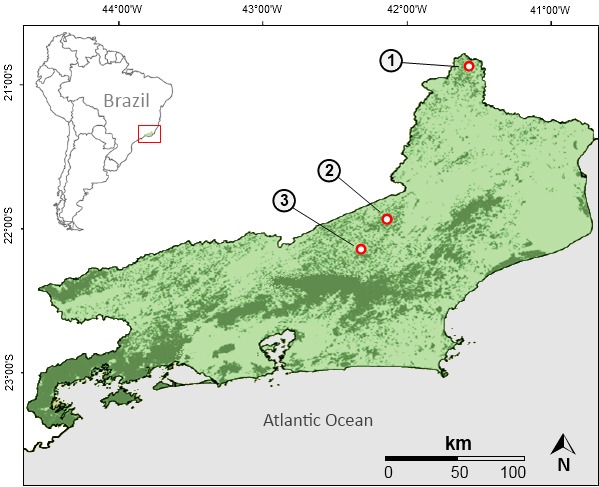
Remnants of Atlantic Forest in the state of Rio de Janeiro (dark green), and location of the three surveyed areas in the municipalities of Varre-Sai (1), Cantagalo (2) and Sumidouro (3).

**Figure 1b.**
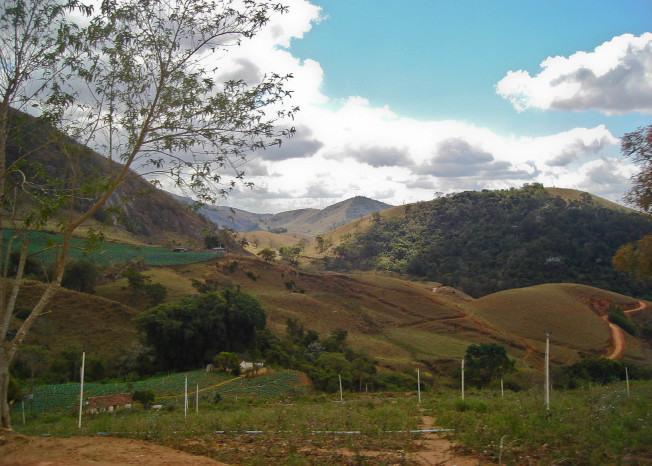
Sampled area in Varre-Sai.

**Figure 1c.**
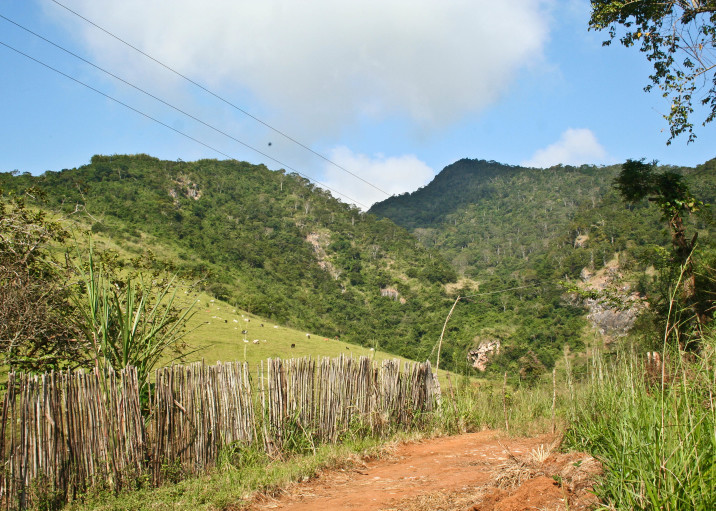
Sampled area in Cantagalo.

**Figure 1d.**
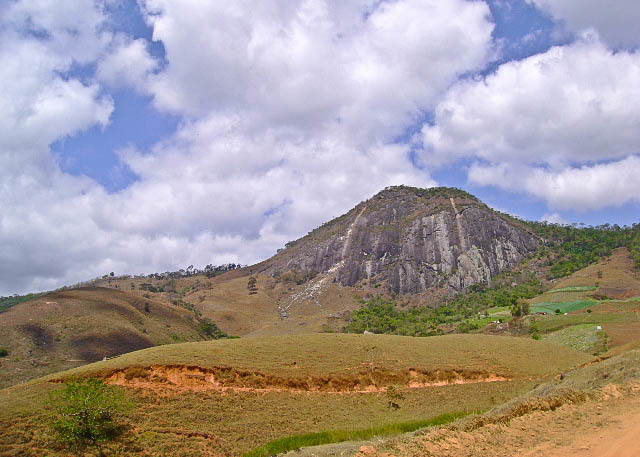
Sampled area in Sumidouro.

**Table 1.** Absolute and relative abundances (%) of bat species, and parameters of the bat community (species richness, dominance, equitability, and diversity) for the three localities studied here.

**Taxa**	**Trophic** **guild**	**Captures**		
		**Sumidouro**	**Cantagalo**	**Varre-Sai**
** Emballonuridae **				
*Peropteryx macrotis*	Insectivore	0	5	0
** Phyllostomidae **				
**Desmodontinae**				
*Desmodus rotundus*	Sanguivore	10	5	0
*Diphylla ecaudata*	Sanguivore	1	2	1
**Glossophaginae**				
*Anoura caudifer*	Nectarivore	21	1	2
*Glossophaga soricina*	Nectarivore	0	1	0
**Carolliinae**				
*Carollia perspicillata*	Frugivore	30	10	22
**Phyllostominae**				
*Chrotopterus auritus*	Carnivore	3	0	0
*Macrophyllum macrophyllum*	Insectivore	1	0	0
**Stenodermatinae**				
*Artibeus fimbriatus*	Frugivore	0	0	2
*Artibeus lituratus*	Frugivore	5	0	1
*Artibeus obscurus*	Frugivore	3	0	0
*Chiroderma doriae*	Frugivore	3	0	0
*Platyrrhinus lineatus*	Frugivore	0	0	1
*Platyrrhinus recifinus*	Frugivore	3	0	0
*Sturnira lilium*	Frugivore	0	0	10
*Vampyressa pusilla*	Frugivore	2	0	1
** Vespertilionidae **				
*Myotis nigricans*	Insectivore	0	1	1
**Total**		**82**	**25**	**41**
Species richness		11	7	9
Capture efficiency (m².h)		0.010	0.005	0.006
Dominance (D')		0.224	0.251	0.355
Equitability (J')		0.761	0.821	0.648
Shannon-Wiener index (H')		1.826	1.599	1.426

**Table 2.** Bat species richness by habitat, considering the three areas combined.

**Species**	**Habitats**			
	**Forest**	**Edge**	**Riparian**	**Open areas**
*Peropteryx macrotis*	0	0	0	5
*Anoura caudifer*	7	2	1	14
*Artibeus fimbriatus*	2	0	0	0
*Artibeus lituratus*	5	1	0	0
*Artibeus obscurus*	3	0	0	0
*Carollia perspicillata*	7	13	19	23
*Chiroderma doriae*	3	0	0	0
*Chrotopterus auritus*	3	0	0	0
*Desmodus rotundus*	10	0	0	5
*Diphylla ecaudata*	1	1	0	2
*Glossophaga soricina*	0	0	0	1
*Macrophyllum macrophyllum*	0	0	1	0
*Platyrrhinus lineatus*	0	0	1	0
*Platyrrhinus recifinus*	3	0	0	0
*Sturnira lilium*	1	8	1	0
*Vampyressa pusilla*	2	1	0	0
*Myotis nigricans*	0	0	1	1
**Total of captures**	**47**	**26**	**24**	**51**
Species richness	12	6	6	7
Capture efficiency (m².h)	0.012	0.006	0.006	0.013
Dominance (D')	0.121	0.355	0.635	0.300
Equitability (J')	0.913	0.715	0.472	0.745
Shannon-Wiener index (H')	2.270	1.282	0.847	1.451

## References

[B959091] Asbjornsen Heidi, Ashton Mark S., Vogt Daniel J., Palacios Sergio (2004). Effects of habitat fragmentation on the buffering capacity of edge environments in a seasonally dry tropical oak forest ecosystem in Oaxaca, Mexico. Agriculture, Ecosystems & Environment.

[B959122] Bergallo Helena Godoy, Esbérard Carlos Eduardo Lustosa, Mello Marco Aurelio Ribeiro, Lins Viviane, Mangolin Renato, Melo Glauce G. S., Baptista Marcia (2003). Bat species richness in Atlantic Forest: what is the minimum sampling effort?. Biotropica.

[B959446] Bergallo H. G., Vicente R. S., Baptista R. L.C., Bomtempo C. B.T., Saraça C. E.S., Baptista D. F., Silva H. R., Salgado N. C., Bergallo H. G., Fidalgo E. C.C., Rocha C. F.D., Uzeda M. C., Costa M. B., Alves M. A.S., Van Sluys M., Santos M. A., Costa T. C.C., Cozzolino A. C.R. (2009). egião Agropecuária dos Rios Pomba, Muriaé e Itabapoana. Estratégias e ações para a conservação da biodiversidade no Estado do Rio de Janeiro.

[B959135] Bernard Enrico, Aguiar Ludmilla M. S., Machado Ricardo B. (2010). Discovering the Brazilian bat fauna: a task for two centuries?. Mammal Review.

[B959474] Delciellos A. C., Novaes Roberto Leonan Morim, Loguercio M. C.F., Geise L., Santori R. T., Souza R. F., Papi B. S., Raíces D., Vieira N. R., Felix S., Detogne N., Souza-da-Silva C. C., Bergallo H. G., Rocha-Barbosa O. (2012). Mammals of Serra da Bocaina National Park, state of Rio de Janeiro, southeastern Brazil. Check List.

[B959145] Dias Daniela, Peracchi Adriano Lúcio (2008). Quirópteros da Reserva Biológica do Tinguá, estado do Rio de Janeiro, sudeste do Brasil (Mammalia: Chiroptera). Revista Brasileira de Zoologia.

[B959494] Dias D., Esbérard Carlos Eduardo Lustosa, Peracchi A. L., Reis N. R., Peracchi A. L., Santos G. A.S. (2008). Riqueza, diversidade de espécies e variação altitudinal de morcegos na Reserva Biológica do Tinguá, estado do Rio de Janeiro, Brasil (Mammalia, Chiroptera). Ecologia de Morcegos.

[B959155] Dias Daniela, Peracchi Adriano Lúcio, Silva Shirley Seixas Pereira (2002). Quirópteros do Parque Estadual da Pedra Branca, Rio de Janeiro, Brasil (Mammalia, Chiroptera). Revista Brasileira de Zoologia.

[B959508] Esbérard Carlos Eduardo Lustosa (2003). Diversidade de morcegos em área de Mata Atlântica regenerada no sudeste do Brasil. Revista Brasileira Zoociências.

[B959165] Esbérard Carlos Eduardo Lustosa (2007). Influência do ciclo lunar na captura de morcegos Phyllostomidae. Iheringia. Série Zoologia.

[B959518] Esbérard Carlos Eduardo Lustosa, Bergallo H. G. (2005). Research on bats in the state of Rio de Janeiro, southeastern Brazil. Mastozoología Neotropical.

[B959175] Esbérard Carlos Eduardo Lustosa, Baptista Márcia, Costa Luciana Moraes, Luz Júlia Lins, Lourenço Elizabete Captivo (2010). Morcegos de Paraíso do Tobias, Miracema, Rio de Janeiro. Biota Neotropica.

[B959528] Esbérard Carlos Eduardo Lustosa, Jordão-Nogueira T., Luz J. L., Melo G. G., Mangolin R., Jucá N., Raíces D. S.L., Enrici M. C., Bergallo H. G. (2006). Morcegos da Ilha Grande, Angra dos Reis, RJ, Sudeste do Brasil. Revista Brasileira de Zoociências.

[B959196] Estrada A., Coates-Estrada R. (2002). Bats in continuous forest, forest fragments and in an agricultural mosaic habitat-island at Los Tuxtlas, Mexico. Biological Conservation.

[B959186] Estrada Alejandro, Coates-Estrada Rosamond, Meritt Dennis (1993). Bat species richness and abundance in tropical rain forest fragments and in agricultural habitats at Los Tuxtlas, Mexico. Ecography.

[B959206] Faria Deborah (2006). Phyllostomid bats of a fragmented landscape in the north-eastern Atlantic forest, Brazil. Journal of Tropical Ecology.

[B959400] Galindo-Leal C, Câmara IG (2005). Mata Atlântica: biodiversidade, ameaças e perspectivas.

[B959216] Gorresen P. Marcos, Miles Adam C., Todd Christopher M., Bonaccorso Frank J., Weller Theodore J. (2008). Assessing bat detectability and occupancy with multiple automated echolocation detectors. Journal of Mammalogy.

[B959227] Guariguata Manuel R, Sáenz Grace P (2002). Post-logging acorn production and oak regeneration in a tropical montane forest, Costa Rica. Forest Ecology and Management.

[B959879] Hammer Ø., Harper D. A.T., Ryan P. D. (2001). PAST: Paleontological statistics software package for education and data analysis. Palaeontologia Electronica.

[B959237] Heithaus E. Raymond, Fleming Theodore H. (1978). Foraging movements of a frugivorous bat, *Carollia
perspicillata* (Phyllostomatidae). Ecological Monographs.

[B959247] Herrera Carlos M., Jordano Pedro, Lopez-Soria Luis, Amat Juan A. (1994). Recruitment of a mast-fruiting, bird-dispersed tree: bridging frugivore activity and seedling establishment. Ecological Monographs.

[B959919] Kalko Elisabeth K. V., Handley Charles O., Handley Darelyn, Cody M. L., Smallwood J. A. (1996). Organization, diversity, and long-term dynamics of a Neotropical bat community. Long-term studies of vertebrate communities.

[B959543] Kunz T. H., Kurta A., Kunz T. H. (1988). Capture methods and holding devices. Ecology and behavioral methods for the study of bats.

[B959257] Luz Júlia Lins, Costa Luciana de Moraes, Lourenço Elizabete Captivo, Esbérard Carlos Eduardo Lustosa (2011). Morcegos (Mammalia, Chiroptera) da Reserva Rio das Pedras, Rio de Janeiro, Sudeste do Brasil. Biota Neotropica.

[B959856] Magurran A. E. (1998). Ecological diversity and its measurement.

[B959267] Medellín Rodrigo A., Equihua Miguel, Amin Miguel A. (2000). Bat diversity and abundance as indicators of disturbance in Neotropical rainforests. Conservation Biology.

[B959277] Meyer Christoph F. J., Weinbeer Moritz, Kalko Elisabeth K. V. (2005). Home-range size and spacing patterns of *Macrophyllum
macrophyllum* (Phyllostomidae) foraging over water. Journal of Mammalogy.

[B959287] Modesto Thiago Carvalho, Pessôa Flávia Soares, Enrici Maria Carlota, Attias Nina, Jordão-Nogueira Tássia, Costa Luciana de Moraes, Albuquerque Hermano Gomes, Bergallo Helena de Godoy (2008). Mamíferos do Parque Estadual do Desengano, Rio de Janeiro, Brasil. Biota Neotropica.

[B959620] Modesto T. C., Pessôa F. S., Jordão-Nogueira T., Enrici M. C., Costa L. M., Attias N., Almeida J., Raíces D. S.L., Albuquerque H. G., Pereira B. C., Esbérard Carlos Eduardo Lustosa, Bergallo H. G. (2008). Mammals, Serra da Concórdia, State of Rio de Janeiro, Brazil. Check List.

[B959586] Moratelli R., Peracchi A. L., Cronemberger C., Viveiros-de-Castro E. B. (2007). Morcegos (Mammalia, Chiroptera) do Parque Nacional da Serra dos Órgãos. Ciência e conservação na Serra dos Órgãos.

[B959301] Murcia Carolina (1995). Edge effects in fragmented forests: implications for conservation. Trends in Ecology & Evolution.

[B959311] Myers N, Mittermeier RA, Mittermeier CG, Fonseca GAB, Kent J (2000). Biodiversity hotspots for conservation priorities.. Nature.

[B959322] Novaes Roberto Leonan Morim, Laurindo Rafael de Souza, Souza Renan de França, Gregorin Renato (2014). Bat assemblage in remnants of Atlantic Forest in Minas Gerais State, southeastern Brazil. Neotropical Biology and Conservation.

[B959687] Peracchi A. L., Nogueira M. R. (2010). Lista anotada dos morcegos do Estado do Rio de Janeiro, sudeste do Brasil. Chiroptera Neotropical.

[B959332] Portfors Christine V., Fenton M. Brock, Aguiar L. M.S., Baumgarten Julio E., Vonhof Maarten J., Bouchard Sylvie, Faria Deborah, Pedro Wagner A., Rauntenbach Naas I. L., Zortea Marlon (2000). Bats from Fazenda Intervales, Southeastern Brazil: species account and comparison between different sampling methods. Revista Brasileira de Zoologia.

[B959056] Ribeiro Milton Cezar, Metzger Jean Paul, Martensen Alexandre Camargo, Ponzoni Flávio Jorge, Hirota Márcia Makiko (2009). The Brazilian Atlantic Forest: How much is left, and how is the remaining forest distributed? Implications for conservation. Biological Conservation.

[B959348] Rocha Patricio Adriano, Mikalauskas Jefferson Simanas, Gouveia Sidney Feitosa, Silveira Victor Vilas-Bôas, Peracchi Adriano Lúcio (2010). Morcegos (Mammalia, Chiroptera) capturados no Campus da Universidade Federal de Sergipe, com oito novos registros para o estado. Biota Neotropica.

[B959359] Sedlock Jodi L., Weyandt Sarah E., Cororan Laura, Damerow Marin, Hwa Shi-Hsia, Pauli Benjamin (2008). Bat diversity in tropical forest and agro-pastoral habitats within a protected area in the Philippines. Acta Chiropterologica.

[B959817] Simmons N. B., Voss R. S. (1998). The mammals of Paracou, French Guiana: a neotropical lowland rainforest fauna Part 1. Bats.. Bulletin of American Museum of Natural History.

[B959409] Soulé ME, Wilcox BA, Soulé ME, Wilcox BA (1980). Conservation biology: its scope and its challenge. Conservation biology.

[B959837] Straube F., Bianconi G. V. (2002). Sobre a grandeza e a unidade utilizada para estimar o esforço de captura com utilização de redes-de-neblin. Chiroptera Neotropical.

[B959423] Tim RM, McDade LA, Bawa KS, Hespenheide HA, Hartshorn GS (1994). The mammals fauna. La Selva: ecology and natural history of a neotropical rain forest.

[B959787] Vizotto L. D., Taddei V. A. (1973). Chave para determinação de quirópteros brasileiros. Revista da Faculdade de Filosofia, Ciências e Letras de São José do Rio Preto.

[B959371] Weinbeer Moritz, Meyer Christoph F. J., Kalko Elisabeth K. V. (2005). Activity pattern of the trawling phyllostomid bat, *Macrophyllum
macrophyllum*, in Panama. Biotropica.

